# Tissue clearing method in visualization of cancer progression and metastasis

**DOI:** 10.48101/ujms.v129.10634

**Published:** 2024-04-09

**Authors:** Kei Takahashi-Yamashiro, Kohei Miyazono

**Affiliations:** a Department of Chemistry, Faculty of Science, University of Alberta, Edmonton, Alberta, Canada; b Department of Applied Pathology, Graduate School of Medicine, The University of Tokyo, Tokyo, Japan; c Laboratory for Cancer Invasion and Metastasis, Institute for Medical Sciences, RIKEN, Yokohama City, Kanagawa, Japan

**Keywords:** cancer metastasis, tissue-clearing, single-cell resolution, 3D imaging, LSFM, EMT

## Abstract

Since various imaging modalities have been developed, cancer metastasis can be detected from an early stage. However, limitations still exist, especially in terms of spatial resolution. Tissue-clearing technology has emerged as a new imaging modality in cancer research, which has been developed and utilized for a long time mainly in neuroscience field. This method enables us to detect cancer metastatic foci with single-cell resolution at whole mouse body/organ level. On top of that, 3D images of cancer metastasis of whole mouse organs make it easy to understand their characteristics. Recently, further applications of tissue clearing methods were reported in combination with reporter systems, labeling, and machine learning. In this review, we would like to provide an overview of this technique and current applications in cancer research and discuss their potentials and limitations.

## Imaging modalities in cancer research

Metastasis is a lethal event for cancer patients, and controlling metastasis is a key action to prolong their survival rates. It has been extensively studied for deciphering how cancer cells metastasize to other organs from primary tumors; however, detailed mechanisms are still not fully understood. To elucidate the complicated mechanisms, it is important to detect the initiation of cancer metastasis *in vivo*. Toward this purpose, various imaging modalities have been developed and utilized for cancer research. For example, computed tomography (CT) and magnetic resonance imaging (MRI) are noninvasive imaging system for cancer diagnosis (1, 2). Bioluminescence imaging (BLI) with luciferase has been widely used to monitor cancer progression and metastasis in mouse models (3, 4). Recent improvement in BLI enables the monitoring of cancer progression spatiotemporally with single-cell resolution (5). Positron emission tomography (PET), nuclear magnetic resonance (NMR), ultra-sound imaging, and fluorescence imaging are also utilized (1, 2, 6). These various imaging systems are accelerating early detection of cancer and leading to improved prognosis; however, there are still limitations, especially in terms of spatial resolution.

## Tissue-clearing methods and microscopies

Tissue-clearing technology has a long history and has been used dominantly in neuroscience research. This technology was first reported over 100 years ago by the German anatomist Walter Spalteholz (7). Then, it has been developed extensively in recent decades, and many types of clearing methods are currently available (8). These were categorized with three groups, including solvent-based (hydrophobic, i.e. BABB and 3DISCO), aqueous-based (hydrophilic, i.e. Sca*l*e, SeeDB, and Ce3D), and hydrogel-based (i.e. CLARITY) (9–17). The basic strategy lying on all methods is minimizing light scattering by removing lipids (delipidation), suppressing light absorption by removing pigments (decolorization), and matching the refractive index (RI). To sustain the tissue structure and molecules, fixation is required before clearing with paraformaldehyde (PFA) or glutaraldehyde (GA), which are commonly used in many protocols (12, 18, 19). Polyepoxide molecules such as polyglycerol-3-polyglycidyl ether (P3PE) are also used for fixation in the stabilization to harsh conditions via intramolecular epoxide linkages to prevent degradation (SHIELD) protocol, which can retain protein, mRNA, and fluorescent protein at high rate after clearing (20). With ethylenediaminetetraacetic acid (EDTA), bones can be cleared by removing calcium phosphate (decalcification) (21, 22). Various methods including 3DISCO and CLARITY are used for clearing, but all methods have pros and cons. Although these methods were categorized into the three groups above, recent studies suggested that combining solvent-based and aqueous-based delipidation would be beneficial, and many researchers reported various combined protocols (23). In 2016, Ueda’s group developed an aqueous-based tissue-clearing method, which is called CUBIC (clear, unobstructed brain/body imaging cocktails, and computational analysis) (21, 24, 25). The CUBIC reagents are safe to handle and can be easily reproduced with high transparency. This method enables us to analyze whole mouse bodies/organs with single-cell resolution (24, 26). Some recent studies succeeded to clear human tissues with high quality. Ertürk and colleagues introduced small-micelle-mediated human organ efficient clearing and labeling (SHANEL), which revealed three-dimensional (3D) structure of human brain, eye, and kidney (27, 28). Staining with antibodies or dye molecules (labeling) is often performed to visualize target molecules with 3D images (29). Labeling with a nanobody instead of an antibody is a new trend considering its high tissue penetration, which is due to the small size of the nanobody (approximately 15 kDa) as it only contains the variable domain of the heavy chain of heavy chain only antibody. Vaughan’s group reported that the FLASH (fast light-microscopic analysis of antibody-stained whole organs) method with mild antigen retrieval restored epitopes and improved sample permeability, resulted in better staining (30, 31).

For visualizing cell structures with cleared samples in 3D, microscopy is required, which can image in deeper tissue with high resolution. Confocal microscopy and two-photon microscopy are options to be selected especially for magnified fields. However, they are incorporated with a point-scanning system, which results in long acquisition time. Light sheet fluorescence microscopy (LSFM) is becoming a very powerful tool for cleared tissues with rapid scanning, which reduces photobleaching (32). Using LSFM, 3D images are reconstituted from whole mouse brains to whole mouse body within a short time (33). Wei et al. showed that Raman scattering (SRS) microscopy can also be used with cleared mouse tissues, resulting in visualization with over 10-fold depth increased (34).

## Utilization of tissue-clearing methods to detect cancer metastasis

**Figure 1:**
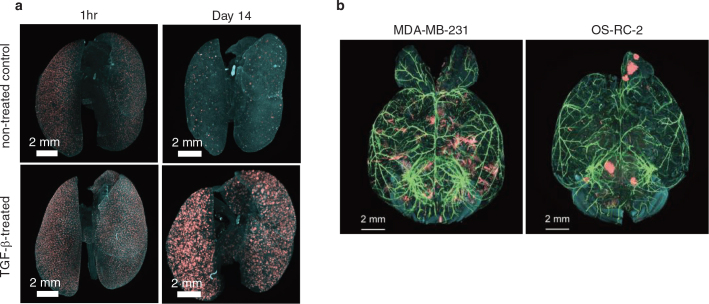
Representative 3D images of cancer metastasis visualized by CUBIC. (a) Lung metastasis after intravenous injection of A549 lung adenocarcinoma cells. A549 cells pretreated with or without TGF-β were injected intravenously, and lungs were analyzed by CUBIC (red: A549 cancer cells expressing mCherry, light blue: nuclear staining with Red-Dot2). (b) Brain metastasis after intracardiac injection of cancer cells. MDA-MB-231 human breast cancer cells or OS-RC-2 human renal cell carcinoma cells were injected intracardially, and brains were analyzed by CUBIC (green: α-SMA^+^ blood vessels labeled with FITC, red: cancer cells expressing mCherry, light blue: nuclear staining with Red-Dot2). The images shown in this figure are from Kubota et al. (18).

Tissue-clearing methods had been used mainly in neuroscience research, and it was just recently that researchers started using them for cancer research. Our group reported the applications of CUBIC with 13 mouse cancer models using 9 cancer cell lines (18). There are two major advantages of this imaging system relative to other imaging modalities. First, cancer metastasis in mouse organs can be detected by this method with single-cell resolution at whole organ level. We succeeded in visualizing lung metastasis foci with single cell resolution (Figure 1A). We studied the effect of transforming growth factor (TGF)-β in cancer metastasis, which is a key cytokine to induce epithelial-mesenchymal transition (EMT) (35). To study the role of TGF-β, we injected human lung adenocarcinoma A549 cells intravenously into mice with or without TGF-β pretreatment. We found out that TGF-β-treated A549 cells have a higher ability to metastasize in lungs (Figure 1A) (18). The quantification results indicated that the EMT phenotype induced in cancer cells did not affect the process of capillary arrest but accelerated survival and proliferation of the cells in the lungs. This observation could be achieved because CUBIC enabled us to count the exact number of cancer cells in whole mouse lung samples. Interestingly, the post-immunohistochemistry after CUBIC revealed that metastatic A549 cells reversed to epithelial type with E-cadherin expression through mesenchymal-epithelial transition (MET), suggesting that plasticity of EMT is necessary for survival and proliferation of cancer cells at metastatic sites (36, 37). This aspect of tissue-clearing methods, that is precise quantification with single cell resolution, could also be applied for the evaluation of antitumor drugs at the whole organ level in mice (18). The second advantage of this imaging system is that cancer metastasis can be monitored with 3D images. In brain metastasis, we clearly distinguished the patterns between cancer cell lines (Figure 1B). MDA-MB-231 human breast cancer cells can only survive along the alpha-smooth muscle actin-positive (α-SMA^+^) blood vessels (Figure 1B) (18). On the other hand, OS-RC-2 human renal cell carcinoma cells showed volumetric metastasis foci far from blood vessels (Figure 1B) (18). These differences were not clear when we analyzed the data with 2D images, suggesting that 3D images give us a valuable insight into characteristics of cancer metastasis. Many other studies showed application of tissue-clearing both in mouse and human organs bearing cancer metastasis (38). Nojima et al. reported that 3D analysis of clinical lymph node (LN) specimens showed higher metastatic detection sensitivity compared to the conventional 2D method (39).

Our group succeeded in capturing 3D images with not only whole mouse organs but also a whole mouse body. Using CUBIC, we showed distribution of cancer metastasis, including spontaneous metastasis to the lung in OS-RC-2 orthotopic inoculation model (18). Ertürk’s group also reported whole mouse body imaging with cancer metastasis in a mouse bearing pancreatic cancer using vDISCO (nanobody(VHH)-boosted 3D imaging of solvent-cleared organs) (40). In addition, his group succeeded in monitoring not only cancer metastasis but also distribution of antitumor antibody 6A10 (anti-CA12 antibody) (41). These studies indicated the important advantage that tissue clearing can give us spatial information of cancer metastasis and kinetics of antibodies at whole mouse body level with single-cell resolution.

**Figure 2:**
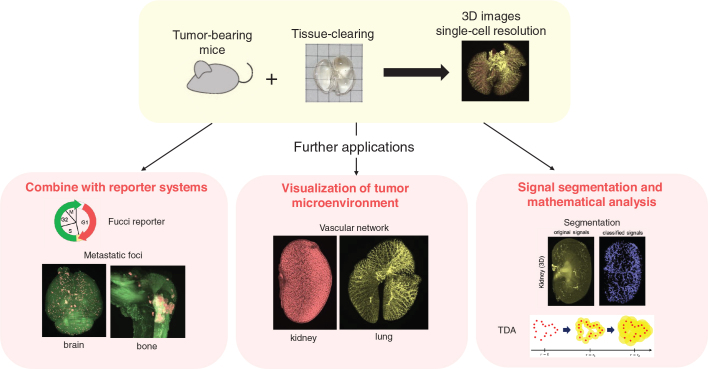
Applications of tissue-clearing in cancer research. After tissue clearing of the organ/body of tumor bearing mice, 3D analyses with single-cell resolution are conducted. In addition, the analyses using tissue-clearing methods can be combined with some other methods, including combination with some reporter systems, visualization of tumor microenvironment, and signal segmentation and mathematical/AI analysis. The images shown in this figure are from Takahashi et al. (42), and Takahashi et al. (43).

Various potential applications of tissue-clearing technology have been explored (Figure 2). For example, it would be beneficial if we can add cell cycle information on top of a spatial information of cancer metastasis. Our study showed cell cycle of cancer cells in each metastatic foci using fluorescent ubiquitination-based cell cycle indicator (Fucci) reporter system developed by Miyawaki and colleagues, which can detect cell cycle by combination of multicolor fluorescent proteins (44–46). Interestingly, the same cell line metastasized to different organs showed heterogeneity with different stages in cell cycle (42). This study also focused on the effect of antitumor drugs on cell cycle and showed that 5-fluorouracil (5-FU) treatment blocked cell cycle at G2/M phase in 4T1 mouse breast cancer cells (42). Some foci, however, showed the 5-FU treated 4T1 cells at G0/G1 phase, and this heterogeneity between tumor foci in the same organ could contribute to antidrug tumor resistance. Heterogeneity of cancer cells also plays a key role when circulating tumor cells make clusters and metastasize to the second organ, which is called polyclonal metastasis (47–49). Kok et al. showed 3D images of liver metastasis injected with intestinal tumor-derived organoids and revealed that non-metastatic cancer cells can metastasize via the polyclonal metastasis through fibrotic change induced by metastatic cancer cells (50). Our group compared the lung metastasis efficiency when mice were injected intravenously with mixture of two different phenotypes of cancer cells, that is TGF-β-treated mesenchymal A549 cells and TGF-β-untreated epithelial A549 cells (51). The result showed that TGF-β-treated A549 cells accelerated metastasis of non-treated A549 cells, indicating that EMT-induced cancer cells changed the characteristics of the tumor microenvironment at metastatic sites and played a role in forming a metastatic niche (51). In the report by Tanaka et al., human and mouse formalin-fixed paraffin-embedded (FFPE) tumors were cleared with iDISCO, and 3D imaging showed the heterogeneity of EMT and angiogenesis inside the tumors (52). These studies indicate that 3D images by tissue-clearing is compatible with analyzing the cancer heterogeneity at whole mouse organ level.

## Visualization of tumor microenvironment with tissue-clearing

Within tumor microenvironment, not only cancer cells but also other types of cells exist, and complicated interactions among them accelerate progression of cancer metastasis (53). Immune cells are key players within the tumor microenvironment. Tissue-clearing methods enable us to visualize mouse immune cells, including brain microglia with Iba1 and platelets with CD42 (51, 54, 55). With Ce3D (clearing-enhanced 3D), CD8^+^ T cells, B cells, and macrophages in mouse LNs were visualized with high quality (17). Stoltzfus et al. introduced the analysis tool “CytoMAP” for visualizing the distribution of dendritic cells (DCs) in murine LNs (56). In tumor specimens, tumor-associated macrophages (TAM) in mouse lung metastasis were examined, and tumor-associated neutrophils (TANs) with CD66b and T cells with CD3 in HNC (head and neck cancer) tumors were visualized with 3D images (57, 58).

Another important advantage of 3D images is that it is beneficial for studying interactions between cancer foci and vasculatures because vascular networks are challenging to be analyzed with 2D images. New pipelines for the visualization of the vascular networks were reported, which can visualize the cerebral vasculature network with 3D images using clearing methods (59–61). In the report by Kirst et al., veins, arteries, and capillaries were visualized by immunolabeling of several markers (i.e. Podocalyxin, CD31, and α-SMA), and datasets were generated from 20 mouse brains with characterization of vascular network across brain areas (59). Our group also succeeded in capturing 3D images of mouse blood and lymphatic vessels by visualizing VE-cadherin, α-SMA, and Prox1 (Prospero homeobox transcription factor 1) and established a new quantification method in combination with one of the mathematical analysis, known as topological data analysis (TDA) (43). Lung lymphatic vessels were visualized in mouse melanoma B16F10 intravenous injection model, and the distances between metastatic foci and lymphatic vessels were successfully calculated with 3D images. The result indicated that lymphatic vessels and cancer cells are getting closer in a time-dependent manner during the formation of metastatic foci. In addition, TDA analysis suggested that the structure of lymphatic vessels might be remodeled after intravenous injection of B16F10 tumor cells (43). Thus, if transcriptomic analysis at single-cell level can be applied to tissue-clearing technology, the roles of lymphatic system in progression of cancer metastasis can be analyzed at the molecular level (see later).

When cancer cells, immune cells, or vasculatures are visualized with high quality, segmentation is required for further quantification. Various methods have been established using machine learning (59, 60, 62). Our group uses semi-automated segmentation using ilastik software for signal classification, and data processing was performed in python as described (43, 51, 54, 63, 64). ImageJ/FIJI and some commercial software including IMARIS are also available for segmentation and counting. Although most organs are difficult to be normalized among samples due to variations in their shape or size, mouse brains are relatively easy to normalize because of their uniformity with respect to size and shape. The 3D data obtained by tissue-clearing can be applied to a reference brain data for normalization such as CUBIC-Atlas, which is originally based on Allen Brain Atlas (54, 65). This type of normalization provides anatomical brain information to help determine precise localization of metastasis in brain areas.

## Limitations and potentials of tissue-clearing for applications in cancer research

Tissue-clearing methods opened the new doors in cancer research, and now cancer metastasis could be monitored with single-cell resolution at whole mouse organ/body level. However, there are some limitations to overcome. First, this method needs fixation before clearing, and therefore, we can only get snapshot data under current protocols. Some efforts have been put into clearing with a live mouse; however, it is not yet feasible. To overcome this, combining tissue-clearing with other imaging modalities such as BLI or CT would play a key role in spatiotemporal monitoring of metastasis, which gives us time-series information. Second, data size becomes larger along with higher quality images captured by LSFM. As denoted before, workstations equipped with large RAM (random access memory) (i.e. 128–256 GB) are required for image processing, including segmentation, quantification, and further mathematical analysis, which can restrict their applications (43). The last point is relevant for conducting further analysis in terms of gene expression. Using tissue-clearing technology, we can capture the exact location of cancer metastasis with single cell resolution. As discussed earlier, combining reporter system, dyes, or staining markers would give us extra information. It would be ideal if gene expression of cancer metastasis is examined simultaneously. Recently, technology for probing spatial transcriptomics has emerged, which gives us gene expression data and 2D spatial information simultaneously (66, 67). These spatial transcriptomic systems (i.e. Visium by 10X Genomics, GeoMX DSP by NanoString) are implemented in many research facilities (68). Although some clearing methods are not compatible for retaining mRNA, optimized tissue-clearing methods can capture mRNA signals with 3D images using mFISH (single-molecule fluorescent in situ hybridization), which is a basic method for some spatial transcriptomic technologies (20, 55, 69, 70). Heinz and Murakami recently reported that their workflow “mFISH3D” enables mRNAs to be visualized using clearing with high resolution in mouse and human brains (71). In terms of proteomics, Ertürk’s group recently succeeded in spatial mass spectrometry from small portion of cleared samples (72). Considering the recent progress in these fields, it might be feasible in the near future to combine tissue-clearing and spatial transcriptomics, which would give us information on exact location and gene expression relevant to cancer metastasis simultaneously with single-cell resolution at whole mouse organ level. After overcoming these drawbacks, we believe that tissue-clearing technology could be a strong imaging modality in cancer research.
